# 4-Cyano­anilinium iodide

**DOI:** 10.1107/S1600536812033466

**Published:** 2012-08-01

**Authors:** Joel T. Mague, David J. Vumbaco, Michael N. Kammer, Lynn V. Koplitz

**Affiliations:** a Department of Chemistry, Tulane University, New Orleans, LA 70118, USA; b Department of Biological Sciences, Loyola University, New Orleans, LA 70118, USA; c Department of Physics, Loyola University, New Orleans, LA 70118, USA; d Department of Chemistry, Loyola University, New Orleans, LA 70118, USA

## Abstract

In the title compound, C_7_H_7_N_2_^+^·I^−^, the cation is located on a site of 4*mm* symmetry and is thus disordered about the fourfold axis so that there are two perpendicular orientations of the six-membered ring and four rotational orientations of the {–NH_3_^+^} group. In the crystal, there are two layers perpendicular to the *c* axis, each containing iodide ions and the {–NH_3_^+^} portions of the cations, with the remainder of the cations extending outwards from these layers.

## Related literature

For the structure of 4-cyano­anilinium chloride, see: Colapietro *et al.* (1981 ▶). For the structure of 4-cyano­anilinium bromide, see: Vumbaco *et al.* (2012 ▶). For the structure of anilinium iodide, see: Fecher & Weiss (1986 ▶).
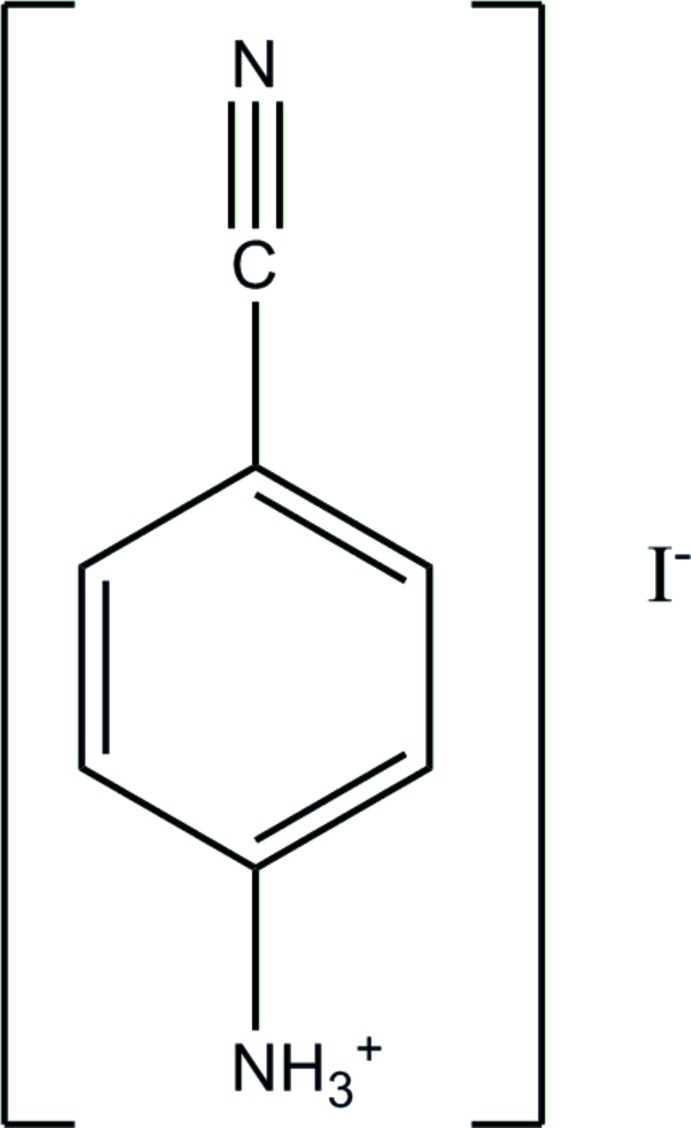


## Experimental

### 

#### Crystal data

C_7_H_7_N_2_^+^·I^−^
*M* *_r_* = 246.05
Tetragonal, 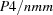

*a* = 4.9930 (4) Å
*c* = 16.445 (2) Å
*V* = 409.98 (8) Å^3^
*Z* = 2
Mo *K*α radiation
μ = 3.83 mm^−1^
*T* = 100 K
0.26 × 0.20 × 0.05 mm

#### Data collection

Bruker SMART APEX CCD diffractometer
Absorption correction: multi-scan (*SADABS*; Sheldrick, 2009 ▶) *T*_min_ = 0.362, *T*_max_ = 0.844
7082 measured reflections
382 independent reflections
381 reflections with *I* > 2σ(*I*)
*R*_int_ = 0.039

#### Refinement

*R*[*F* ^2^ > 2σ(*F* ^2^)] = 0.012
*wR*(*F* ^2^) = 0.029
*S* = 1.14
382 reflections
31 parameters
H-atom parameters constrained
Δρ_max_ = 0.46 e Å^−3^
Δρ_min_ = −0.56 e Å^−3^

### 

Data collection: *APEX2* (Bruker, 2010 ▶); cell refinement: *SAINT* (Bruker, 2009 ▶); data reduction: *SAINT*; program(s) used to solve structure: *SHELXS97* (Sheldrick, 2008 ▶); program(s) used to refine structure: *SHELXL97* (Sheldrick, 2008 ▶); molecular graphics: *SHELXTL* (Sheldrick, 2008 ▶); software used to prepare material for publication: *SHELXTL*).

## Supplementary Material

Crystal structure: contains datablock(s) I, global. DOI: 10.1107/S1600536812033466/rk2373sup1.cif

Structure factors: contains datablock(s) I. DOI: 10.1107/S1600536812033466/rk2373Isup2.hkl

Supplementary material file. DOI: 10.1107/S1600536812033466/rk2373Isup3.cml

Additional supplementary materials:  crystallographic information; 3D view; checkCIF report

## Figures and Tables

**Table 1:** Hydrogen-bond geometry (Å, °)

*D*—H⋯*A*	*D*—H	H⋯*A*	*D*⋯*A*	*D*—H⋯*A*
N1—H1⋯I1	0.88	2.72	3.5813 (5)	165
N1—H1*A*⋯I1^i^	0.88	2.87	3.5813 (5)	139
N1—H1*B*⋯I1^ii^	0.88	2.87	3.5813 (5)	139

Symmetry codes: (i) 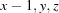; (ii) 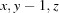.

**Fig. 1.**
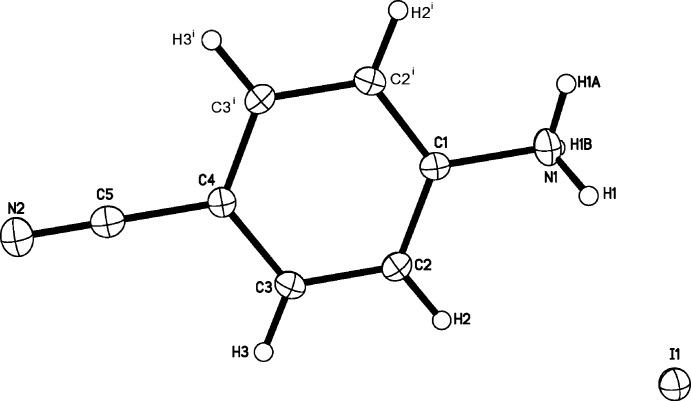
View of the cation–anion pair with with the atom numbering scheme. Displacement ellipsoids are drawn at 50% probability level. H atoms are presented as a small spheres of arbitrary radius. Symmetry code: (i) -x+1/2, -y+1/2, z.

**Fig. 2.**
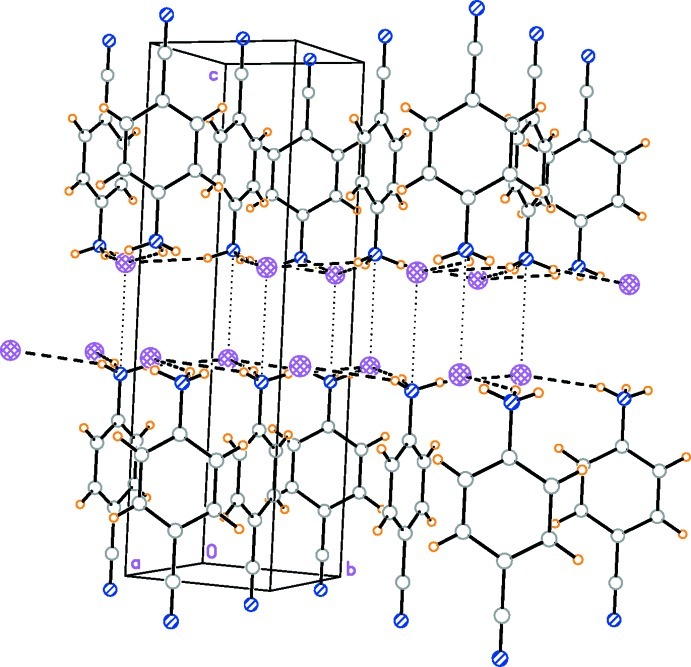
Packing showing the cation–anion bilayer perpendicular to the c axis. N—H···I interactions are shown as dashed lines and the electrostatic N+···I- interactions as dotted lines. Colour key: purple = I, blue = N, gray = C, orange = H.

## supplementary crystallographic information

### Comment

In the title compound, (C_7_H_7_N_2_)^+^.I^-^, the cation is located on a site of 4*mm* symmetry so is disordered over 2 sites rotated 90° from one another about the N1—C1···C4—C5≡N2 axis. This leads to a more extensive disorder of the (—NH_3_^+^) group so it is likely that there are a variety of N—H···I interactions of different geometries. To illustrate what one set of these could be, the best estimate of the rotational orientation of the (—NH_3_^+^) group obtained from a difference map was used to generate the values given in Table 1. These interactions lead to a layer of anions with the (—NH_3_^+^) groups largely in the layer and the remainder of the cations projecting perpendicular to the layer. Two of these layers are then associated in a head–to–head fashion *via* electrostatic N1^+^···I1^-^ interactions of 3.513 (1)Å leading to a bilayer of iodide and (—NH_3_^+^) groups with the remainders of the cations projecting out from both sides (Fig. 2). A similar layer structure is adopted by anilinium iodide (Fecher & Weiss, 1986) while the packings for 4–cyanoanilinium bromide (Vumbaco *et al.*, 2012) and the corresponding chloride salt (Colapietro *et al.*, 1981) are quite different.

### Experimental

The 0.55 g of 4–cyanoaniline and 1.0 ml of aqueous hydroiodic acid (47% by mass) were combined in 10 ml of ethanol. This solution was slowly evaporated to dryness under ambient conditions to form crystals of the title compound.

### Refinement

The cation sits on a special position requiring 4 mm symmetry with the 4–fold axis running through both N atoms and the attached carbons. Thus the carbon atoms at the 2– and 3–positions are effectively disordered over two sites and two orientations of each were used in the refinement. H atoms attached to these carbons were placed in calculated positions with C—H = 0.95Å. A small peak in a position to be one location for a hydrogen bound to N1 could be seen in a difference map and its position was used to calculate positions for the remainder of one of the orientations of the —NH_3_^+^ unit in which the N—H distance was adjusted to be 0.88Å. All H atoms were included as riding contributions with *U*_iso_(H) = 1.2*U*_eq_(C, N).

### Crystal data

**Table:**

C_7_H_7_N_2_^+^·I^−^	*D*_x_ = 1.993 Mg m^−^^3^
*M**_r_* = 246.05	Mo *K*α radiation, λ = 0.71073 Å
Tetragonal, *P*4/*n**m**m*	Cell parameters from 8495 reflections
Hall symbol: -P 4a 2a	θ = 2.5–29.1°
*a* = 4.9930 (4) Å	µ = 3.83 mm^−^^1^
*c* = 16.445 (2) Å	*T* = 100 K
*V* = 409.98 (8) Å^3^	Plate, colourless
*Z* = 2	0.26 × 0.20 × 0.05 mm
*F*(000) = 232	

### Data collection

**Table:**

Bruker SMART APEX CCD diffractometer	382 independent reflections
Radiation source: fine–focus sealed tube	381 reflections with *I* > 2σ(*I*)
Graphite monochromator	*R*_int_ = 0.039
φ and ω scans	θ_max_ = 29.1°, θ_min_ = 2.5°
Absorption correction: multi-scan (*SADABS*; Sheldrick, 2009)	*h* = −6→6
*T*_min_ = 0.362, *T*_max_ = 0.844	*k* = −6→6
7082 measured reflections	*l* = −22→22

### Refinement

**Table:**

Refinement on *F*^2^	Primary atom site location: heavy atom
Least-squares matrix: full	Secondary atom site location: difference Fourier map
*R*[*F*^2^ > 2σ(*F*^2^)] = 0.012	Hydrogen site location: inferred from neighbouring sites
*wR*(*F*^2^) = 0.029	H-atom parameters constrained
*S* = 1.14	*w* = 1/[σ^2^(*F*_o_^2^) + (0.0165*P*)^2^ + 0.1447*P*] where *P* = (*F*_o_^2^ + 2*F*_c_^2^)/3
382 reflections	(Δ/σ)_max_ = 0.002
31 parameters	Δρ_max_ = 0.46 e Å^−^^3^
0 restraints	Δρ_min_ = −0.56 e Å^−^^3^

### Special details

**Table:**

Experimental. The diffraction data were obtained from 3 sets of 400 frames, each of width 0.5° in ω, colllected at φ = 0.00°, 90.00° and 180.00° and 2 sets of 800 frames, each of width 0.45° in φ, collected at ω = -30.00° and 210.00°. The scan time was 10 sec/frame.
Geometry. All s.u.'s (except the s.u. in the dihedral angle between two l.s. planes) are estimated using the full covariance matrix. The cell s.u.'s are taken into account individually in the estimation of s.u.'s in distances, angles and torsion angles; correlations between s.u.'s in cell parameters are only used when they are defined by crystal symmetry. An approximate (isotropic) treatment of cell s.u.'s is used for estimating s.u.'s involving l.s. planes.
Refinement. Refinement of *F*^2^ against ALL reflections. The weighted *R*–factor *wR* and goodness of fit *S* are based on *F*^2^, conventional *R*–factors *R* are based on *F*, with *F* set to zero for negative *F*^2^. The threshold expression of *F*^2^ > σ(*F*^2^) is used only for calculating *R*–factors(gt) *etc*. and is not relevant to the choice of reflections for refinement. *R*–factors based on *F*^2^ are statistically about twice as large as those based on *F*, and *R*–factors based on ALL data will be even larger.

### Fractional atomic coordinates and isotropic or equivalent isotropic displacement parameters (Å^2^)

**Table:**

	*x*	*y*	*z*	*U*_iso_*/*U*_eq_	Occ. (<1)
I1	0.7500	0.7500	0.588552 (10)	0.01472 (8)	
N1	0.2500	0.2500	0.62508 (16)	0.0199 (5)	
H1	0.3662	0.3662	0.6057	0.024*	0.25
H1A	0.0887	0.2904	0.6073	0.024*	0.125
H1B	0.2904	0.0887	0.6073	0.024*	0.125
N2	0.2500	0.2500	1.03941 (17)	0.0226 (6)	
C1	0.2500	0.2500	0.71467 (18)	0.0139 (5)	
C2	0.4934 (6)	0.2500	0.75539 (17)	0.0174 (5)	0.50
H2	0.6576	0.2500	0.7262	0.021*	0.50
C3	0.4936 (6)	0.2500	0.84022 (16)	0.0171 (5)	0.50
H3	0.6583	0.2500	0.8692	0.020*	0.50
C4	0.2500	0.2500	0.88189 (18)	0.0143 (5)	
C5	0.2500	0.2500	0.97006 (19)	0.0174 (6)	

### Atomic displacement parameters (Å^2^)

**Table:**

	*U*^11^	*U*^22^	*U*^33^	*U*^12^	*U*^13^	*U*^23^
I1	0.01513 (9)	0.01513 (9)	0.01392 (11)	0.000	0.000	0.000
N1	0.0236 (8)	0.0236 (8)	0.0126 (11)	0.000	0.000	0.000
N2	0.0252 (9)	0.0252 (9)	0.0174 (12)	0.000	0.000	0.000
C1	0.0129 (8)	0.0129 (8)	0.0158 (12)	0.000	0.000	0.000
C2	0.0132 (12)	0.0225 (14)	0.0163 (11)	0.000	0.0020 (10)	0.000
C3	0.0129 (12)	0.0226 (13)	0.0157 (11)	0.000	−0.0022 (11)	0.000
C4	0.0146 (8)	0.0146 (8)	0.0136 (13)	0.000	0.000	0.000
C5	0.0161 (9)	0.0161 (9)	0.0200 (14)	0.000	0.000	0.000

### Geometric parameters (Å, º)

**Table:**

N1—C1	1.473 (4)	C2—C3	1.395 (4)
N1—H1	0.8800	C2—H2	0.9500
N1—H1A	0.8800	C3—C4	1.396 (3)
N1—H1B	0.8800	C3—H3	0.9500
N2—C5	1.141 (4)	C4—C3^i^	1.396 (3)
C1—C2^i^	1.388 (3)	C4—C5	1.450 (4)
C1—C2	1.388 (3)		
			
C1—N1—H1	111.2	C1—C2—H2	120.8
C1—N1—H1A	109.4	C3—C2—H2	120.3
H1—N1—H1A	109.4	C2—C3—C4	119.3 (3)
C1—N1—H1B	109.4	C2—C3—H3	120.1
H1—N1—H1B	109.4	C4—C3—H3	120.5
H1A—N1—H1B	108.0	C3^i^—C4—C3	121.2 (3)
C2^i^—C1—C2	122.3 (3)	C3^i^—C4—C5	119.39 (15)
C2^i^—C1—N1	118.85 (16)	C3—C4—C5	119.39 (15)
C2—C1—N1	118.85 (16)	N2—C5—C4	180.000 (1)
C1—C2—C3	118.9 (3)		
			
C2^i^—C1—C2—C3	0.000 (2)	C1—C2—C3—C4	0.000 (2)
N1—C1—C2—C3	180.000 (1)	C2—C3—C4—C5	180.000 (1)

Symmetry code: (i) −*x*+1/2, −*y*+1/2, *z*.

### Hydrogen-bond geometry (Å, º)

**Table:**

*D*—H···*A*	*D*—H	H···*A*	*D*···*A*	*D*—H···*A*
N1—H1···I1	0.88	2.72	3.5813 (5)	165
N1—H1*A*···I1^ii^	0.88	2.87	3.5813 (5)	139
N1—H1*B*···I1^iii^	0.88	2.87	3.5813 (5)	139

Symmetry codes: (ii) *x*−1, *y*, *z*; (iii) *x*, *y*−1, *z*.
